# Photochemical isomerization reactions of acrylonitrile. A mechanistic study†

**DOI:** 10.1039/c7ra12614j

**Published:** 2018-02-02

**Authors:** Ming-Der Su

**Affiliations:** Department of Applied Chemistry, National Chiayi University Chiayi 60004 Taiwan; Department of Medicinal and Applied Chemistry, Kaohsiung Medical University Kaohsiung 80708 Taiwan midesu@mail.ncyu.edu.tw

## Abstract

The mechanisms for the photochemical isomerization reactions are determined theoretically using the acrylonitrile model molecule. The CASSCF (twelve-electron/eleven-orbital active space) and MP2-CAS methods are respectively used with the 6-311G(d,p) and 6-311++G(3df,3pd) basis sets. The structure of the conical intersection that plays a prominent role in the photoisomerization of acrylonitrile is obtained. The intermediates and the transition structures of the ground states are also calculated, to allow a qualitative explanation of the reaction pathways. These model studies suggest that the preferred reaction route is: acrylonitrile → Franck–Condon region → conical intersection → isoacrylonitrile → transition state → intermediate complex → transition state → cyanoacetylene. The theoretical evidence suggests that conical intersections found in this paper can give a better understanding of the photochemical reactions of acrylonitrile and support the experimental observations.

## Abstract 2

The computational findings indicate that conical intersection plays a central role in understanding the photochemical behaviors of acrylonitrile.

## Graphical abstract

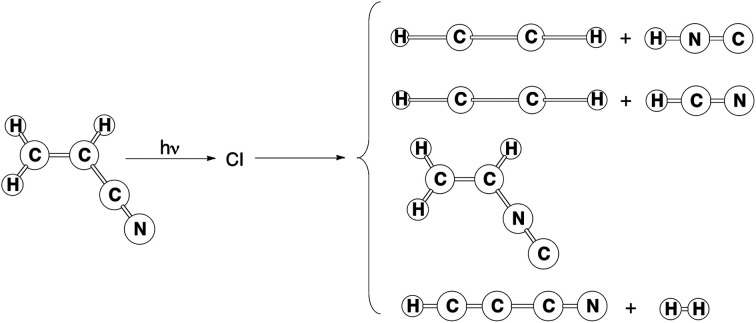

## I. Introduction

Nitrile chemistry, which is found in a variety of astrochemical environments, is involved in diverse areas of astronomy and planetary science and has played a dominant role in the analysis of many interstellar observations.^1^ In particular, acrylonitrile (vinylcyanide, cyanoethylene, propenenitrile, H_2_C=C(H)CN) has been detected in Titan's atmosphere and in molecular clouds.^2^ Therefore, acrylonitrile derivatives have attracted the attention of many experimental and theoretical chemists because of their abundance in the interstellar medium, their unusual conjugated structures and their rich photochemistry.^3,4^

**Scheme 1:**
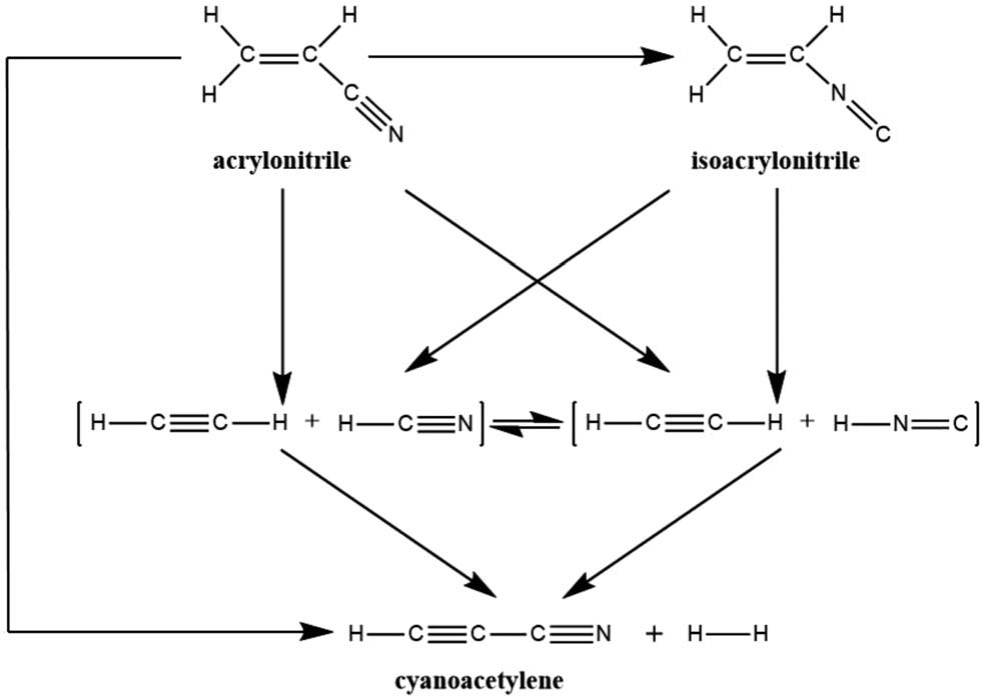
Proposed photolysis pathways of acrylonitrile.

Recently, Couturier-Tamburelli, Piétri and co-workers made a photochemical study of acrylonitrile,^5,6^ which was trapped in low-temperature argon matrices and then irradiated using a microwave discharge hydrogen-flow lamp (*λ* > 120 nm = 10.3 eV). Several photoproducts were identified: (H_2_C=C(H)NC) (isoacrylonitrile), HC_3_N (cyanoacetylene), C_2_H_2_:HCN (acetylene:hydrogen cyanide), and C_2_H_2_:HNC (acetylene:hydrogen isocyanide) complexes.^5^ These have all been detected in molecular clouds or in Titan's atmosphere.^2–4^ As a result of these photochemical results, Couturier-Tamburelli, Piétri and co-workers provided the reaction scheme for the photolysis products gained by irradiation of H_2_C=C(H)CN at *λ* > 120 nm, which is shown in Scheme 1. In this proposed photolysis scheme, however, there is no consideration of the photoreaction pathway from the excited state to the ground state. As a result, this may raise several unanswered mechanistic questions. For instance, what is the real photolysis mechanism, from which one may have a clear mechanistic picture to understand the photochemical behaviour of acrylonitrile?

It is the above experimental results and unsolved problems that arouse our interest to investigate its photolysis mechanism. To the best of the author's knowledge, several papers reported by different laboratories^4^ have been considered the similar chemical reactions. Nevertheless, these studies only discussed the potential energy surface for the unimolecular ground-state dissociation of acrylonitrile. That is to say, they did not regard the reaction pathway from the excited state to the ground state, needless to mention the formation pathway of isoacrylonitrile. As a result, using a more sophisticated quantum chemical theory is necessary to examine photochemical reactions of acrylonitrile (Scheme 1). Indeed, all photochemical reactions start on an excited potential surface, but cross over to a lower surface somewhere along the reaction pathway. Subsequently, they arrive at the ground-state surface through a series of radiation-less transitions (*i.e.*, conical intersections; CIs). Then, they finally move on the ground-state surface towards the product.^7^ Since the above experimental evidences^5,6^ did not report any radiation transitions, this strongly indicates that a direct crossover from the excited state to the ground state hypersurface occurs. As a consequence, CIs should play a crucial role in the photolysis reactions of acrylonitrile. In this work, the CI intersection region on the potential surfaces has been located where decay to the ground-state surface can occur and ground-state reaction paths that lead from the CI to a variety of products have been identified. It is hoped that these theoretical investigations will give a better understanding of the thermodynamic and kinetic aspects of these acrylonitrile photoreactions and will allow optimal explanations for further interstellar observations.

## II. Methodology

The computational results at the *ab initio* CASSCF level of theory were performed using the Gaussian 09 software package.^8^ For the study of the present photochemical reaction pathways, the twelve electrons in eleven orbitals^9^ CASSCF method was used with the 6-311G(d,p) basis sets, for geometry optimization. The optimization of CIs was achieved in the (*f* − 2)-dimensional intersection space using the method of Bearpark *et al.*^10^ that is implemented in the Gaussian 09 program. Minima and transition states (TSs) were confirmed by calculating the harmonic vibrational frequencies along the reaction-path. Since it has been reported that the addition of high-exponent *d* and *f* inner polarization functions is essential to obtaining reliable energies for second-row species in most cases,^11^ the 6-311++G(3df,3pd) basis sets were used. To correct the energetics for dynamic electron correlation, single-point calculations were made at the MP2-CAS-(12,11)/6-311++G(3df,3pd) level, using CAS(12,11)/6-311G(d,p) geometry (depicted in MP2//CASSCF).^12^ Unless otherwise stated, the relative energies given are determined at the MP2 level.

## III. Results and discussion

**Fig. 1:**
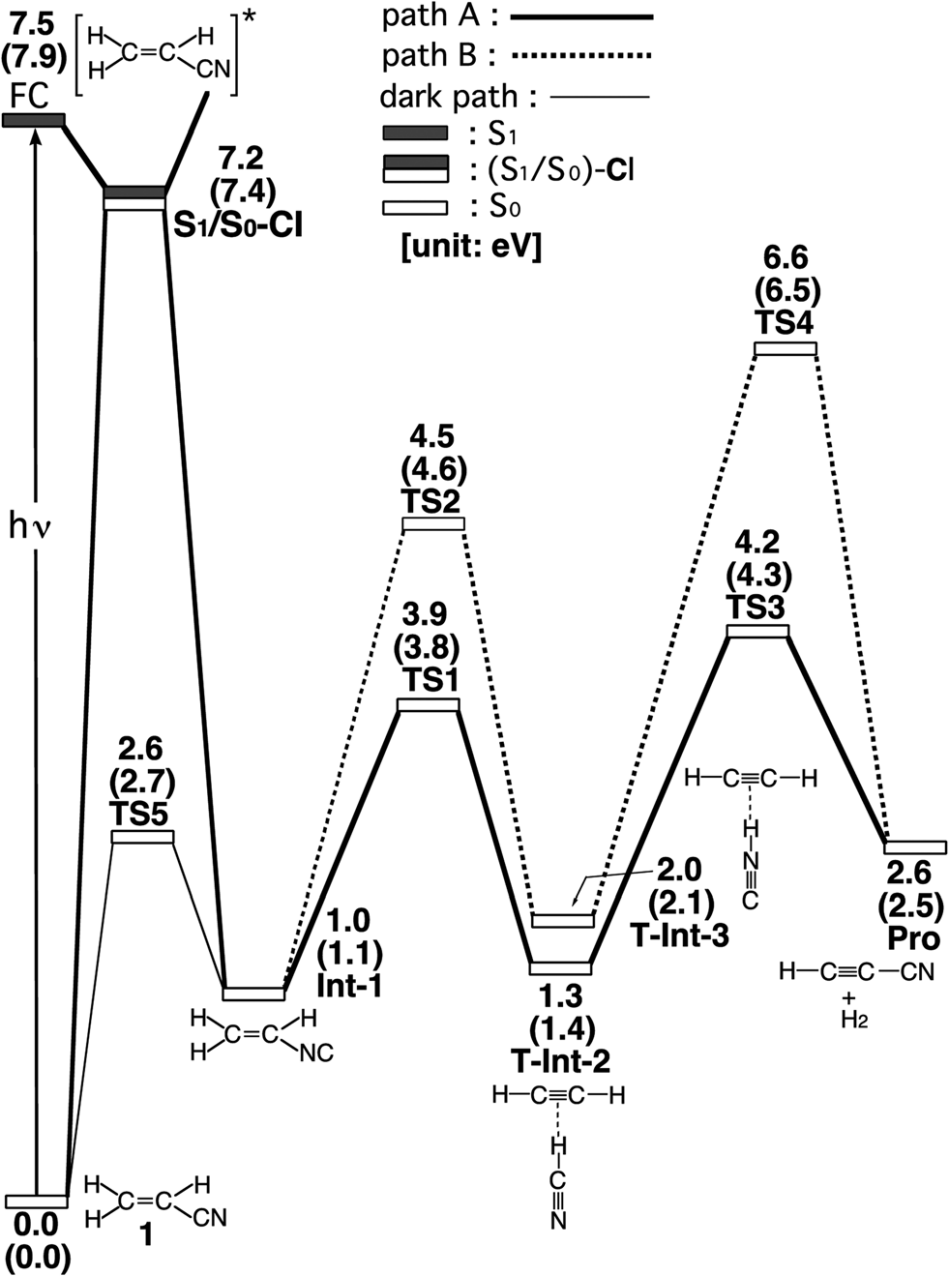
Energy profiles for the photoisomerization reactions of acrylonitrile (1). The abbreviations FC, CI, Int, TS, and Pro stand for Frank–Condon, conical intersection, intermediate, transition state, and product, respectively. The relative energies were obtained at the CAS(12,11)/6-311G(d,p) (in parentheses) and MP2-CAS-(12,11)/6-311++G(3df,3pd)//CAS(10,7)/6-311G(d,p) levels of theory. All energies (in eV) are given with respect to the reactant (1). The CASSCF optimized structures of the crucial points see Fig. 2. For more information see the text.

**Fig. 2:**
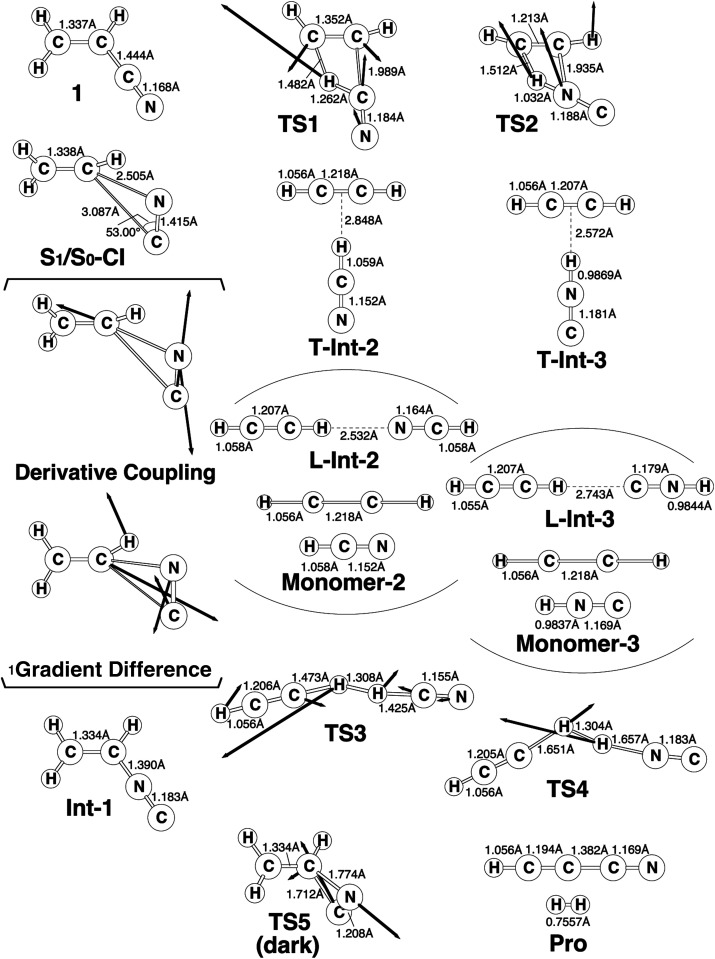
The CAS(12,11)/6-311G(d,p) geometries (in Å and deg) for path A and path B of acrylonitrile (1), conical intersection (CI), intermediate (Int), transition state (TS), and isomer product (Pro). The derivative coupling and gradient difference vectors—those which lift the degeneracy—computed with CASSCF at the conical intersection S_1_/S_0_-CI. The corresponding CASSCF vectors and the imaginary frequency describing the different TSs are shown inset. For more information see the text.

The experimental studies^5^ characterize the two minimum-energy pathways on the singlet excited potential energy surface of acrylonitrile that lead to the same products (cyanoacetylene + hydrogen; Pro) (1): path A and path B. In order to understand the differences between the two reaction paths, it is best to begin with the reaction profiles, which are summarized in Fig. 1. This figure also contains the relative energies of all of the critical points with respect to the energy of the reactant 1. Selected optimized geometrical parameters for the stationary points and conical intersections are shown in Fig. 2. The Cartesian coordinates and the energetics calculated for the various points at the CASSCF level are available as ESI.†

In the first step, acrylonitrile (1) is promoted to its excited singlet state by a vertical excitation, as shown in the left-hand side of Fig. 1. After the vertical excitation process, 1 is situated on the excited singlet surface, but still possesses the S_0_ (ground-state) geometry (FC). The calculated singlet vertical excitation energy (1 → FC) is predicted to be about 7.5 eV. This value is in reasonable agreement with the experimental observations.^5,6^

From the FC point, the excited 1 goes to S_1_/S_0_-CI, where the photoexcited system decays non-radiatively to S_0_. The computational results at the MP2-CAS level of theory predict that the energy of S_1_/S_0_-CI lies 7.2 eV above 1 and 0.3 eV below FC. Funneling through the S_1_/S_0_-CI point can lead to two possible reaction paths on the ground-state surface, *via* either the derivative coupling vector or the gradient difference vector.^7^ The results shown in Fig. 2 clear show that the gradient difference vector is mainly related to the rotation of a C≡N triple bond that gives the doubly bonded intermediate, Int-1 (isoacrylonitrile), on the S_0_ surface, but the derivative coupling vector results in the C≡N bond stretching motion that can lead to a vibrationally hot 1-S_0_ species. The MP2-CAS results also show that the energy of Int-1 lies about 6.5 eV below that of FC. This implies that the large excess energy of 6.5 eV that results from the relaxation from FC to Int-1 is the driving force for further isomerization reactions of 1 on the ground state.

With regard to the structure of Int-1, the search for transition states on the S_0_ surface gives TS-1 and TS-2 for reaction path A and path B, respectively. For path A, a T-shaped intermediate, T-Int-2,^13^ occurs *via* a transition state (TS-1) that involves an intramolecular 1,2-hydrogen shift and a C≡N triple bond rotation. However, for path B, one hydrogen atom from ethylene is inserted into the C–N single bond (TS-2), to form the other T-shaped intermediate, T-Int-3.^13^ It should be noted that three types of intermediates that are related to TS-1 and TS-2 are studied by using the CASSCF method: L-Int-2,^14^Monomer-2, and L-Int-3,^14^Monomer-3. Their geometrical structures are shown in Fig. 2. Regardless of the structures, the MP2-CAS calculations indicate that the structures with the HCN moiety are 0.5–0.8 eV more stable than those with the HNC pattern. In particular, the MP2-CAS results show that the relative energies (eV) decrease in the order: Monomer-2 (1.6) > L-Int-2 (1.3) ≈ T-Int-2 (1.3) and Monomer-3 (2.4) > L-Int-3 (2.0) ≈ T-Int-3 (2.0). As a result, on the basis of these computations, it is predicted that T-Int-2 and T-Int-3 should be experimentally observable, since both are proved to be the most stable intermediates during the respective formation of C_2_H_2_:HCN and C_2_H_2_:HNC complexes formations. The experimental findings^5^ allowed Couturier-Tamburelli, Piétri and co-workers to conclude that the two intermediate complexes (C_2_H_2_:HCN and C_2_H_2_:HNC) adopt a T-shaped structure, which is in agreement with the results of this theoretical work. These MP2-CAS computations also indicate that the activation energies for the Int-1 → TS-1 → T-Int-2 and Int-1 → TS-2 → T-Int-3 processes are respectively estimated to be 2.8 and 3.5 eV. Consequently, because of the large excess energy of 6.5 eV that arises from the relaxation from FC to Int-1, the barrier heights of TS-1 and TS-2 can be expected to be easily overcome to obtain the T-Int-2 and T-Int-3 intermediates.

Beginning from the T-Int-2 point, isoacrylonitrile (Int-1) can then produce cyanoacetylene and hydrogen (Pro) *via* the TS-3 point (path A). From the T-Int-3 intermediate, the 1 molecule can also yield the same final products (Pro) *via* the TS-4 state (path B). Fig. 1 shows that the energies of the TS-3 and TS-4 states that connect T-Int-2 and Pro and T-Int-3 and Pro on the S_0_ surface respectively lie 4.2 and 6.6 eV above that of the reactant, 1. The final products, Pro, are also higher in energy by 2.6 eV, compared with acrylonitrile 1. Again, the excess energy of molecule 1 has is about 6.5 eV, which arises from the relaxation from FC to Int-1. This energy is greater than the respective energy barriers of TS-3 (2.9 eV) and TS-4 (4.6 eV), for paths A and B. This theoretical evidence shows that this molecular system has sufficient internal energy to overcome these energy barriers and yield the final products (Pro). Therefore, these theoretical findings are in good agreement with the experimentally verified fact that acrylonitrile can easily undergo intramolecular rearrangement to produce various kinds of photoproducts (*i.e.*, isoacrylonitrile, acetylene:hydrogen cyanide, acetylene:hydrogen isocyanide, and cyanoacetylene), after photo absorptions.^5^

In short, the photolysis mechanism for the singlet photochemical reaction of acrylonitrile 1 can be represented as follows:

Path A:1(S_0_) + *hν* → FC → Int-1 → TS-1 → T-Int-2 → TS-3 → Pro

Path B:1(S_0_) + *hν* → FC → Int-1 → TS-2 → T-Int-3 → TS-4 → Pro

The dark (thermal) reaction on the ground-state potential energy surface is also studied in this work. In spite of the fact that photo-excitation promotes 1 into an excited electronic state, the product of the photochemical reaction is controlled by the ground-state potential surface.^7^ As a result, the search for transition states on the ground-state surface near the structure of S_1_/S_0_-CI gives TS5. Its computational structure is given in Fig. 2. Fig. 1 shows that for the dark (thermal) reaction, the energy of TS5, which connects 1 and Int-1 on the S_0_ surface, lies 4.6 eV below the energy of the S_1_/S_0_-CI. It is noteworthy that the MP2-CAS results indicate that the energy barriers for 1 → Int-1 and Int-1 → 1 are respectively predicted to be 2.7 and 1.6 eV. This theoretical evidence suggests that it would be difficult to produce the doubly bonded isoacrylonitrile (Int-1) *via* the thermal (dark) reaction, let alone the further isomerization reactions on the S_0_ surface that yield the various products detailed. However, since these computational results demonstrate that the energy barrier for Int-1 → 1 is significantly smaller (1.6 eV) than the excess energy (6.5 eV) when it has decayed through a conical intersection point, isoacrylonitrile should easily return to reactant 1 on the ground state.

## VI. Conclusion

The reaction mechanisms for the photoreaction of acrylonitrile (1) are studied with respect to the formation of various types of photoproducts. It should be noted that this study provides the first theoretical demonstration of dynamics simulations, a theoretical estimation of the activation energy and the reaction enthalpy for the photochemical processes.

These theoretical observations suggest that the C–N bond of acrylonitrile is rotated when the molecule absorbs a photon, to reach an excited state *via* a singlet transition, which decreases the energy gap between the ground state energy surface and the energy surface of the first excited state. As a result, on the basis of the present theoretical evidences, this excited molecule presumably enters an extremely efficient decay channel, which takes the form of a conical intersection between the excited- and ground-state potential energy surfaces. After decay at the conical intersection point, acrylonitrile continues to make the CN moiety rotation, followed by recombination with a C_2_H_3_ unit, to yield isoacrylonitrile (Int-1) on the ground state potential surface. Because of the large excess internal energy from the FC point to isoacrylonitrile that results, all energy barriers on the S_0_ surface can be surmounted, to produce various chemical products (acetylene:hydrogen cyanide, acetylene:hydrogen isocyanide, and cyanoacetylene) or revert to the reactant, acrylonitrile. Accordingly, the mechanism studied in this work, which features a conical intersection, gives a better understanding of the photochemical reactions of acrylonitrile and supports the experimental observations.^5,6^

## Conflicts of interest

There are no conflicts to declare.

## Supplementary Material

RA-008-C7RA12614J-s001
