# Supplementary material for: Photochemical isomerization reactions of acrylonitrile. A mechanistic study
Source: RSC Adv. 2018 Feb 2;8(11):5647–51. doi: 10.1039/c7ra12614j (PMC9078173; doi:10.1039/c7ra12614j)
Supplement: RA-008-C7RA12614J-s001 [file RA-008-C7RA12614J-s001.pdf]

Electronic Supplementary Material (ESI) for RSC Advances.
This journal is © The Royal Society of Chemistry 2018

Supplementary Materials

Photochemical Isomerization Reactions of Acrylonitrile. A Mechanistic Study

Ming-Der Su[1,2]*

[1]Department of Applied Chemistry, National Chiayi University, Chiayi 60004, Taiwan

[2]Department of Medicinal and Applied Chemistry, Kaohsiung Medical University, Kaohsiung 80708, Taiwan

*E-mail: midesu@mail.ncyu.edu.tw

(1) **1 (Acrylonitrile)**

Atomic Number	Coordinates (Angstroms) X	Y	Z
1	0.000000	0.000000	0.000000
6	0.000000	0.000000	1.073894
6	1.150698	0.000000	1.754928
1	2.113382	0.000000	1.228753
1	-0.953532	0.000000	1.568559
6	1.206300	0.000000	3.198030
7	1.251278	0.000000	4.365393

CASSCF(12,11)/6-311G(d,p) = -169.974955478
MP2(12,11)/6-311++G(3df,3pd)//CASSCF(12,11)/6-311G(d,p)= -169.9951085

(2) **FC**

CASSCF(12,11)/6-311G(d,p) = -169.6845266
MP2(12,11)/6-311++G(3df,3pd)//CASSCF(12,11)/6-311G(d,p)= -169.7200179

(3) **S1/S0-CI**

Atomic Number	Coordinates (Angstroms) X	Y	Z
1	-0.402997	-0.717679	0.029489
6	-0.275306	0.108375	0.702642
6	0.738983	0.187611	1.565099
1	1.545826	-0.312469	2.020291
1	-0.995719	0.908705	0.674658
6	0.893847	0.556914	4.626453
7	1.661599	-0.330225	3.835883

Derivative Coupling

-1	-0.0006894774	0.0009277660	0.0020458626
-2	0.0020082252	-0.0019575224	0.0025365995
-3	-0.0074703749	0.0035887462	-0.0151570861
-4	-0.0008835638	0.0024543504	0.0014335271
-5	0.0025017349	-0.0006002592	-0.0003041061
-6	0.0240038720	-0.0270279516	-0.0219132153
-7	-0.0194704159	0.0226148706	0.0313584183

Unscaled Gradient Difference

-1	0.0002594426	-0.0010290998	-0.0019055180
-2	-0.0010438218	0.0015574800	0.0002869697

-3	0.0028929569	0.0013704353	0.0113490933
-4	0.0021279596	-0.0048366878	-0.0078011024
-5	-0.0017242863	0.0004352130	-0.0002449467
-6	0.0009325623	-0.0015164745	-0.0029801961
-7	-0.0034448133	0.0040191339	0.0012957002

CASSCF(12,11)/6-311G(d,p) = -169.7010426
MP2(12,11)/6-311++G(3df,3pd)//CASSCF(12,11)/6-311G(d,p) = -169.7311529

(4) **Int-1 (isoacrylonitrile)**

Atomic Number	Coordinates (Angstroms) X	Y	Z
1	0.000000	0.000000	0.000000
6	0.000000	0.000000	1.072912
6	1.155747	0.000000	1.738709
1	2.129673	0.000000	1.238284
1	-0.944837	0.000000	1.582242
7	1.220509	0.000000	3.126897
6	1.275653	0.000000	4.308941

CASSCF(12,11)/6-311G(d,p) = -169.9335427
MP2(12,11)/6-311++G(3df,3pd)//CASSCF(12,11)/6-311G(d,p)= -169.9547482

(5) **TS1**

Atomic Number	Coordinates (Angstroms) X	Y	Z
1	0.000000	0.000000	0.000000
6	0.000000	0.000000	1.059648
6	1.054224	0.000000	1.906249
1	2.018133	0.300297	2.244455
1	-0.474040	0.198484	2.449639
6	-0.057036	-0.308010	3.527387
7	-0.731580	-0.445024	4.490621

-1	0.40	0.50	0.14
-2	-0.12	-0.06	0.05
-3	0.00	-0.04	0.10
-4	0.08	0.31	0.04
-5	-0.08	0.38	-0.52
-6	0.06	-0.02	-0.07
-7	0.02	0.02	-0.05

CASSCF(12,11)/6-311G(d,p) = -169.83597251

MP2(12,11)/6-311++G(3df,3pd)//CASSCF(12,11)/6-311G(d,p)= -169.8531529

(6) **TS2**

	Atomic Number				Coordinates (Angstroms) X	Y	Z
	1				0.000000	0.000000	0.000000
	6				0.000000	0.000000	1.060081
	6				0.385291	0.262777	2.179750
	1				0.843161	0.459367	3.107144
	1				-0.988648	-0.458756	2.108033
	7				-1.133834	-0.466376	3.129892
	6				-1.341899	-0.523473	4.298116
-1	-0.60	0.29	0.01				
-2	-0.01	0.14	0.02				
-3	0.02	-0.05	0.04				
-4	0.06	-0.30	0.04				
-5	0.08	-0.53	-0.01				
-6	0.08	-0.26	-0.03				
-7	-0.06	0.26	-0.03				

CASSCF(12,11)/6-311G(d,p) = -169.8055265
MP2(12,11)/6-311++G(3df,3pd)//CASSCF(12,11)/6-311G(d,p)= -169.8265658

(7) **HCCH + HCN (monomer)**

Atomic Number	Coordinates (Angstroms) X	Y	Z
1	0.000000	0.000000	0.000000
6	0.000000	0.000000	1.055679
6	0.000000	0.000000	2.273363
1	0.000000	0.000000	3.329041

1	0.000000	0.000000	0.000000
6	0.000000	0.000000	1.057614
7	0.000000	0.000000	2.209371

CASSCF(12,11)/6-311G(d,p) = -169.9197985
MP2(12,11)/6-311++G(3df,3pd)//CASSCF(12,11)/6-311G(d,p)= -169.9377115

(8) **HCCH + HCN (linear)**

Atomic Number	Coordinates (Angstroms) X	Y	Z

1	0.000000	0.000000	0.000000
6	0.000000	0.000000	1.058326
7	0.000000	0.000000	2.221852
1	0.000000	0.000000	4.753376
6	0.000000	0.000000	5.811454
6	0.000000	0.000000	7.018159
1	0.000000	0.000000	8.073619

CASSCF(12,11)/6-311G(d,p) = -169.9222299
MP2(12,11)/6-311++G(3df,3pd)//CASSCF(12,11)/6-311G(d,p)= -169.9455159

(9) **HCCH + HCN (T-shape; T-int-2)**

Atomic Number	Coordinates (Angstroms) X	Y	Z
6	-0.001000	0.000000	0.609255
1	2.846769	0.000000	0.000000
6	0.000000	0.000000	-0.609255
1	0.000000	0.000000	-1.665501
1	0.000000	0.000000	1.665501
6	3.905719	0.000000	0.000000
7	5.057726	0.000000	0.000000

CASSCF(12,11)/6-311G(d,p) = -169.9225599
MP2(12,11)/6-311++G(3df,3pd)//CASSCF(12,11)/6-311G(d,p)= -169.9455159

(10) **TS3**

Atomic Number	Coordinates (Angstroms) X	Y	Z
1	0.000000	0.000000	0.000000
6	0.000000	0.000000	1.055702
6	0.000000	0.000000	2.262035
1	0.134531	0.114621	3.724807
1	-0.736076	0.190855	4.697688
6	-0.965043	0.515147	6.066232
7	-1.190678	0.758736	7.172602

-1	0.10	-0.08	0.14
-2	0.03	0.00	0.14
-3	-0.10	0.05	0.14
-4	-0.47	-0.03	-0.78
-5	0.16	0.00	0.16
-6	0.08	-0.03	-0.10
-7	0.00	-0.01	-0.12

CASSCF(12,11)/6-311G(d,p) = -169.7754481

MP2(12,11)/6-311++G(3df,3pd)//CASSCF(12,11)/6-311G(d,p)= -169.7984015

(11) **HCC(CN) + H2**

Atomic Number	X	Y	Z
1	0.000000	0.000000	0.000000
6	0.000000	0.000000	1.055681
6	0.000000	0.000000	2.249202
6	0.000000	0.000000	3.631560
7	0.000000	0.000000	4.800493
1	0.000000	0.000000	0.000000
1	0.000000	0.000000	0.755697

Coordinates (Angstroms)

CASSCF(12,11)/6-311G(d,p) = -169.8805359
MP2(12,11)/6-311++G(3df,3pd)//CASSCF(12,11)/6-311G(d,p) = -169.8997115

(12) **HCCH + HNC (monomer)**

Atomic Number	X	Y	Z
1	0.000000	0.000000	0.000000
6	0.000000	0.000000	1.055679
6	0.000000	0.000000	2.273363
1	0.000000	0.000000	3.329041
1	0.000000	0.000000	0.000000
7	0.000000	0.000000	0.983676
6	0.000000	0.000000	2.152315

Coordinates (Angstroms)

CASSCF(12,11)/6-311G(d,p) = -169.8887559
MP2(12,11)/6-311++G(3df,3pd)//CASSCF(12,11)/6-311G(d,p)= -169.9077115

(13) **HCCH + HNC (linear)**

Atomic Number	X	Y	Z
1	0.000000	0.000000	0.000000
7	0.000000	0.000000	0.984382
6	0.000000	0.000000	2.163775
1	0.000000	0.000000	4.906940
6	0.000000	0.000000	5.964342
6	0.000000	0.000000	7.171013

Coordinates (Angstroms)

1	0.000000	0.000000	8.225609

CASSCF(12,11)/6-311G(d,p) = -169.8955773
MP2(12,11)/6-311++G(3df,3pd)//CASSCF(12,11)/6-311G(d,p)= -169.9214689

(14) **HCCH + HNC (T-shape; T-int-3)**

Atomic .	Coordinates (Angstroms)		
Number	X	Y	Z
6	0.000000	0.000000	0.603417
1	2.571855	0.000000	0.000000
6	0.000000	0.000000	-0.603417
1	0.000000	0.000000	-1.659208
1	0.000000	0.000000	1.659208
7	3.558805	0.000000	0.000000
6	4.739413	0.000000	0.000000

CASSCF(12,11)/6-311G(d,p) = -169.896582
MP2(12,11)/6-311++G(3df,3pd)//CASSCF(12,11)/6-311G(d,p)= -169.9224676

(15) **TS4**

Atomic .	Coordinates (Angstroms)		
Number	X	Y	Z
1	0.000000	0.000000	0.000000
6	0.000000	0.000000	1.055839
6	0.000000	0.000000	2.260992
1	0.213163	0.000002	3.897840
1	-0.819304	0.000003	4.687785
7	-1.888340	0.000005	5.953845
6	-2.538358	0.000006	6.941883

-1	0.00	0.00	-0.01
-2	0.00	0.00	-0.01
-3	0.00	0.00	-0.02
-4	0.06	0.00	0.31
-5	0.63	0.00	-0.71
-6	-0.03	0.00	0.03
-7	-0.02	0.00	0.03

CASSCF(12,11)/6-311G(d,p) = -169.9225599
MP2(12,11)/6-311++G(3df,3pd)//CASSCF(12,11)/6-311G(d,p)= -169.7520363

(16) **Pro**

Atomic .	Coordinates (Angstroms)		
Number	X	Y	Z

1	0.000000	0.000000	0.000000
6	0.000000	0.000000	1.055681
6	0.000000	0.000000	2.249202
6	0.000000	0.000000	3.631560
7	0.000000	0.000000	4.800493

1	0.000000	0.000000	0.000000
1	0.000000	0.000000	0.755697

CASSCF(12,11)/6-311G(d,p) = -169.8805359
MP2(12,11)/6-311++G(3df,3pd)//CASSCF(12,11)/6-311G(d,p)= -169.8587115

(17) **TS5 (dark)**

Atomic Number	.	Coordinates (Angstroms) X	Y	Z
1		0.000000	0.000000	0.000000
6		0.000000	0.000000	1.072876
6		1.157232	0.000000	1.737224
1		2.123677	0.011187	1.282436
1		-0.944313	-0.069230	1.577677
7		1.147166	-0.825620	3.307666
6		1.260571	0.373093	3.405101

-1	-0.02	-0.27	-0.04
-2	0.00	0.03	-0.03
-3	0.02	0.42	-0.06
-4	0.00	0.27	-0.11
-5	-0.01	0.12	-0.03
-6	0.01	-0.23	0.54
-7	-0.03	-0.19	-0.52

CASSCF(12,11)/6-311G(d,p) = -169.8753205
MP2(12,11)/6-311++G(3df,3pd)//CASSCF(12,11)/6-311G(d,p)= -169.8968823
